# The Use of Time to Pregnancy for Estimating and Monitoring Human Fecundity From Demographic and Health Surveys

**DOI:** 10.1097/EDE.0000000000001296

**Published:** 2020-11-30

**Authors:** Niels Keiding, Mohamed M. Ali, Frank Eriksson, Thabo Matsaseng, Igor Toskin, James Kiarie

**Affiliations:** From the a Section of Biostatistics, Department of Public Health, Faculty of Health Sciences, University of Copenhagen. Copenhagen K, Denmark; b Department of Sexual and Reproductive Health and Research (SRH), World Health Organization, Geneva, Switzerland; c Reproductive Medicine Unit, Stellenbosch University/Tygerberg Academic Hospital, Cape Town, South Africa.

**Keywords:** Current duration, Demographic and Health Surveys, Infertility, Low and middle income countries, Time-to-pregnancy

## Abstract

**Background:**

Available studies on the prevalence of infertility have proved to have certain limitations, with a scarcity of population-based studies and inconsistent reporting from surveys in countries at all income levels. We wanted to test the applicability of the current duration approach to data from the important Demographic and Health Surveys (DHS) program, funded by USAID since its inception in 1985, https://dhsprogram.com/.

**Methods:**

The *current duration approach* assumes that there is a well-defined *time of initiation* of attempts to get pregnant and defines the *current duration* of a still ongoing pregnancy attempt as the time interval from initiation to interview. The DHS interviews do not have an explicit question about initiation. We focused on nullipari and substituted date of “establishment of relationship with current partner” for initiation. Our study used the current duration approach on 15 datasets from DHS during 2002–2016 in eight different countries from sub-Saharan Africa, Asia, and Latin America.

**Results:**

Well-established statistical techniques for current duration data yielded results that for some countries postulated surprisingly long median times to pregnancy and surprisingly high estimates of infertility prevalence. Further study of the data structures revealed serious deviations from expected patterns, in contrast to our earlier experience from surveys in France and the United States where participants were asked explicitly about time of initiation of attempts to become pregnant.

**Conclusions:**

Using cohabitation as a proxy for the initiation of attempts to get pregnant is too crude. Using the current duration approach with DHS data will require more explicit questions during the DHS interviews about initiation of pregnancy attempt.

## Abstract 2

Supplemental Digital Content is available in the text.

The WHO clinical definition (https://icd.who.int/browse11/l-m/en#/http%3a%2f) of female infertility is “a disease of the reproductive system characterized by the failure to achieve a clinical pregnancy after 12 months or more of regular unprotected sexual intercourse.” The impact of infertility includes marriage disruptions, emotional and physical abuse, and extramarital affairs with devastating consequences including depression, injuries, and sexually transmitted infections such as HIV.^1^ In addition to underreporting the true burden of infertility and thus limiting access to care, assisted reproductive technology (ART) and other services are still not widely available due to high costs.^2^ Accurate estimates of infertility prevalence are lacking and as a result the magnitude and the burden of infertility is undetermined leaving many women and couples, particularly those living in low- and middle-income countries (LMICs) suffering the most. A Demographic and Health Surveys (DHS) Comparative Report in 2004 estimated that more than 186 million ever-married women of reproductive age in developing countries attempting conception failed, translating into one in every four couples between ages 15 and 49.^3^ Illustrating the central role of definition of infertility we mention that Mascarenhas et al.^4^ conducted a systematic analysis of 227 surveys and estimated the global level of primary infertility defined as the absence of a live birth for women who desire a child and have been in a union for at least 5 years, during which they have not used any contraceptives to be 1.9%.

Infertility is not considered a priority in many LMICs. The most often used arguments against ART are overpopulation, other health priorities (e.g., family planning, vaccinations, malaria, and HIV), limited government budgets, and limited experience of providers with inadequate facilities capable of sophisticated procedures.^5^ Furthermore, in some LMICs, ART is considered to be expensive and moderately effective, with risks of complications, and unknown effects on women and their offspring.^5^ Experts have argued that fertility treatment should be accessible in LMIC, as women in these countries are vulnerable and face being stigmatized and rejected by society, and the development of feasible low-cost ART initiatives is urgently needed.

Available studies on the prevalence of infertility and estimates of the infertility burden have proved to have certain limitations, such as inconsistent reporting from demographic and health surveys both in LMIC and high-income countries.^6^ Scarcity of population-based studies and the inconsistent definitions of infertility used by demographers and epidemiologists in studies are another major challenges in generating reliable estimates of infertility prevalence.^7^ A systematic review by Gurunath et al.^7^ concluded that it is not possible to synthesize reliable infertility prevalence estimates because of incomparable definitions used; however, they recommended a clinically relevant definition based on the duration of trying for pregnancy coupled with female age. This article is an evaluation of one possible implementation of this approach to study international variations in fertility.

Studies on time to pregnancy (TTP) suggest that the method is simple, but the findings may be affected by methodological assumptions; these include high recall bias with retrospective reports and expense and unfeasiblility of prospective cohorts in resource restricted settings, which may only capture couples planning a pregnancy and miss those not intending to be pregnant.^8,9^ In principle, TTP can be measured prospectively, although in practice, starting observation precisely at initiation is challenging, so most prospective studies will allow appropriately controlled delayed entry, as in a large internet-based such design.^10^ A commonly used design is retrospective observation of TTP, but this suffers the obvious difficulty that only successful attempts are recorded. A third possibility, based on the current duration (CD) of pregnancy attempt, evaluates couples with exposure to pregnancy at the time of the interview and determines their current length of time-at-exposure of pregnancy risk.^11,12^ This approach utilizes a cross-sectional study design, including all couples with exposure to pregnancy risk regardless of prior fertility history or pregnancy intentions.^12,13^ There are two important issues with this approach: first, it assumes the existence of a well-defined date of initiation of pregnancy attempt as well as a method of obtaining that information from the couple. Positive practical experience on this issue was obtained in France by Slama et al.^14,15^ and in the United States by Thoma et al.,^13^ who both included a specific question on the date of initiation in their surveys. Second, the sampling of times to pregnancy via CDs oversamples long TTPs which requires attention in the mathematics underlying the estimation procedures; these matters were central to the methodology papers (e.g., Keiding et al.^12,16^) and will be reviewed in the Methods section.

In a recent study Polis et al.,^17^ using the CD approach and standard information available from DHS data from the Nigeria 2013 DHS survey to generate a population-based TTP distribution and estimate infertility prevalence, reported the estimated median TTP among nulliparous women at a risk of pregnancy as 5.1 months (95% confidence interval [CI]: 4.2, 6.3) and the estimated percentage of infertile couples as 31.1% (95% CI: 27.9%, 34.7%). These authors also concluded that their analysis suggests that information from DHS data permits calculation of infertility prevalence but may need improvement or modification.^17^ Based on the findings of the study by Polis et al.^17^ and the need to generate reliable and accurate infertility prevalence estimates particularly in LMIC, the aim of this article is to estimate population-based TTP and the prevalence of infertility using the CD approach applied on standard DHS surveys from selected LMICs. Specifically, to assess the general validity of this approach for the specific purpose of evaluating fecundity for the nullipara women based on current specifications of the DHS questionnaire. The CD approach is rather novel, and we therefore include a brief introduction to this technique. We have positive experience with this method from studies in France^15^ and the United States^13,18^ based on directly targeted surveys. The question was whether it was feasible to reuse surveys planned for other purposes, and here, we needed to develop modifications and approximations of the methods, not all of which were necessarily justified in the new context.

## DATA

### Note on Ethical Review

We only used fully anonymized data in the public domain.

To avoid complexities related to secondary fertility, we focused on women with no previous children *(nullipari*) and we strived to include a reasonably broad selection of LMIC.

We have used data from DHS that cover four World Health Organization (WHO) regions: African Region (Benin, Nigeria, Senegal, and Tanzania); SouthEast Asian Region (Indonesia); Western Pacific Region (Philippines); and Region of the Americas (Dominican Republic, Colombia).^19^ We strived to include a reasonably broad selection of LMIC, using the crude birth rates for fertility classification. We examined all available surveys in the selected eight countries and excluded surveys with fewer than 200 eligible respondents (nine out of 24 surveys) and retained 15 surveys (Table 1).

**TABLE 1.** Steps of Inclusion Criteria and Final Analysis Sample

Survey	N	S1	S2	S3	S4	S5	S6
Benin 2006	17,794	14,564	12,709	7,403	4,160	2,930	250
Colombia 2009	53,521	38,006	36,262	15,682	14,010	1,823	452
Colombia 2016	38,718	27,557	26,441	10,701	9,542	1,183	316
Dominican Republic 2002	23,384	18,298	17,164	6,765	6,234	1,138	243
Dominican Republic 2007	27,195	20,841	19,723	6,626	6,087	991	245
Indonesia 2002	29,483	25,158	23,447	20,031	17,465	4,984	1,005
Indonesia 2007	32,895	28,212	26,300	22,031	18,753	5,289	1,076
Indonesia 2012	45,607	36,036	34,061	22,480	18,825	5,221	1,183
Nigeria 2008	33,385	26,525	23,263	13,456	9,598	7,789	670
Nigeria 2013	38,948	30,447	26,242	15,153	11,832	9,222	756
Philippines 2003	13,633	10,565	9,783	5,993	4,873	1,844	315
Philippines 2008	13,594	10,443	9,747	5,415	4,596	1,528	271
Senegal 2005	14,602	11,321	10,076	3,856	2,939	2,447	234
Senegal 2010	15,688	12,611	11,408	4,796	3,567	2,956	248
Tanzania 2015	13,266	10,482	9,425	4,793	3,981	2,016	211

The stepwise elimination process leading to the samples used for analysis. Women not fulfilling criteria S1–S6 at interview are eliminated step by step.

N indicates total sample; S1, aged between 18 and 44 years old; S2, not currently pregnant; S3, married once, and currently in union and cohabiting; S4, sexually active (had sex in last 4 weeks); S5, not using any method of contraception; S6, nulliparous.

We retained respondents in the analysis if they were “at risk” of conception at the time of the survey defined as: between 18 and 44 years of age; at the time of the survey, having reported only one partner, currently married or living with a partner; sexually active (as measured by reporting having had sex in the last 4 weeks); never had a live birth (nulliparous), menstruating and not currently pregnant, not menopausal, had not had hysterectomy, and was not contracepting at the time of interview (we return in the discussion to the difficulties associated to only recording use of contraception at interview). We did not have access to information on possible infertility treatment for these women. The outcome variable is the CD of risk, defined as the difference between the initiation of cohabitation and the date of the interview, measured in months. Table 1 surveys the stepwise inclusion process for each of the 15 datasets.

## THE CURRENT DURATION APPROACH TO ANALYZING TIME-TO-PREGNANCY

The original proposal^11^ of what is now termed the CD design for estimating the distribution of TTP

would involve a population-based random sample of women who are asked whether they are currently at risk of becoming pregnant. Those who are sexually active and not contracepting are then asked how many cycles have passed since they discontinued contraception, and may be questioned about exposures of interest.

Using TTP assumes the somewhat idealized view that there is a well-defined time of initiation (the moment when the couple discontinues contraception) and that attempts are made without serious breaks until either success (pregnancy) or a time where the couple give up trying. (We ignore for the present the important issue of handling fertility treatment.)

The CD of a still ongoing pregnancy attempt is the time interval from initiation to interview. Practical use of this as realized in France^14,15^ and the United States^13^ followed Weinberg and Gladen in assuming that time of initiation can be retrieved at a retrospective interview, with a specific question either about the date of initiation or directly about the elapsed length so far of the pregnancy attempt.

Thoma et al.,^13^ using data from the United States, explicitly concluded that “Infertility prevalence based on a CD approach was consistent with other US prospective cohort studies with preconception enrolment.” Slama et al.^15^ gave a detailed discussion of the compatibility of their results to several other European studies using various designs, either the popular retrospective pregnancy-based design which excludes infertile women or the historically prospective design.

The appropriate analysis of the collected CDs requires attention to the implicit length-biased selection: longer TTPs have larger chance of being included in the sample. This is handled in the statistical methods literature, see for example Keiding et al.^12,16^ Basically, under the assumption that initiations happen at a constant rate over time, it is intuitively clear and easily proved mathematically that the probability density of the distribution over time of CDs is proportional to the survivor function (that is, 1-distribution function) of TTP. There are several proposals as to estimate this distribution from observed CDs. Here, we focus on two parametric distributions: the Pareto distribution and a more flexible distribution, particularly discussed by Yamaguchi.^20^ In addition, we have used two proposals for nonparametric estimates, the classical nonparametric maximum likelihood estimator (MLE) first derived by Grenander^21^ and the recent smoothed nonparametric MLE (SMLE) proposed by Groeneboom and Jongbloed.^22,23^

**FIGURE 1.**
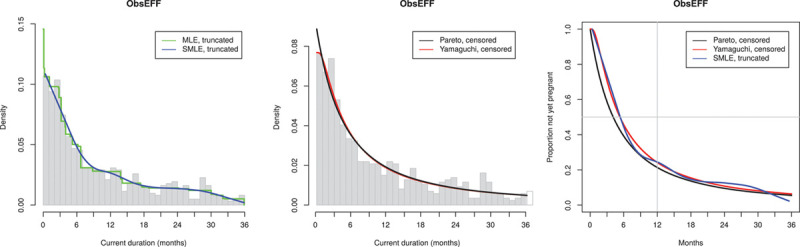
Reanalysis of data from French telephone survey ObsEFF^15^ (867 observations, of which 618 < 36 months). A: Nonparametric fits of CD distribution; B: Parametric fits of CD distribution; C: Estimated distribution of TTP.

We illustrate the approach reanalyzing the data from the French telephone survey ObsEFF,^15^ see Figure 1. In this illustration, we restrict attention to the common standard^13–15,17^ of analyzing CDs censored (for nonparametric fits: truncated) at 36 months. For further explanation of censoring and truncation, see the exemplification below based on the present data.

The results of the reanalysis of the ObsEFF data are shown in Figure 1. Figure 1A shows the histogram (truncated at 36 months) of the observed CD, with nonparametric fits to the observed CD as specified above (Grenander MLE, truncated and Groeneboom SMLE, truncated); Figure 1B shows the parametric fits to the observed CD (Pareto, censored at 36 months and Yamaguchi, censored at 36 months); and Figure 1C shows the consequent estimates of the survival function of TTP for all of these except the MLE which is known to be biased. We note that the various estimation methods agree nicely. Using the Yamaguchi fit to the censored data, the estimated median TTP is 5.35 months and “infertility” (probability of not having succeeded in getting pregnant in 12 months) is estimated as 24%.

## PREGNANCY RECOGNITION BIAS

It is well known that pregnancy will usually be discovered with some delay, and this affects the estimation of the TTP distribution. Weinberg et al.,^24^ discussing prospective and retrospective approaches to TTP analysis, looked at this problem for comparing the effect of several exposures on TTP—one will still get unbiased results under the assumption that the recognition delay is similar for all exposures. Slama et al.^14^ noted that the same comments relate to the CD approach.

In the present context, the focus is, however, on the estimation of the absolute distribution of TTP for which delayed recognition will generate a bias.

Polis et al.^17^ used a modification of the observed CD derived by assuming that all pregnancies are discovered exactly 3 months after they happened and carrying through the calculations by


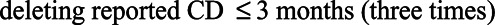


and using


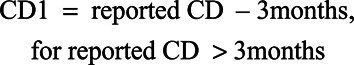


Polis et al. only motivated this procedure by reference to a classical paper by Goldman and Westoff,^25^ which is about obtaining reliable direct counts of the frequency of pregnant women—not directly relevant to the CD approach.

We evaluated the approach by Polis et al. by simple simulations, based on CDs derived from 1 million simulated Weibull distributed TTP with scale parameter 0.33 and shape parameter 0.67, corresponding to mean 6.91 months and SD 10.57 months, with initiation uniformly distributed across 36 to 0 months before the inclusion interview. eFigure 1a; http://links.lww.com/EDE/B742 illustrates that this correction is adequate under the restrictive assumption of a fixed recognition delay of 3 months, whereas eFigure 1b; http://links.lww.com/EDE/B742 shows that the correction is inadequate if recognition delay is more variable. Our conclusion is that the approach to pregnancy bias by Polis et al. does not solve the problem and we have not included it into our calculations.

## MAIN STUDY: COMPARISON OF 15 DATASETS FROM DHS IN EIGHT DIFFERENT COUNTRIES, EACH DATASET CONTAINING AT LEAST 200 PARTICIPANTS

### The Use of Initiation of Relationship as Proxy for Initiation of Attempt to Get Pregnant

A major difficulty with using DHS interviews is that they do not explicitly inquire about initiation of pregnancy attempt. For nullipara, Polis et al.^17^ substituted date of “cohabitation with current partner” for initiation, and we shall use the same definition as specified below. (A detailed comparison of our choices in the analysis compared to those of Polis et al. are in the Online Supplementary Material; http://links.lww.com/EDE/B742.) This substitution carries with it several assumptions that:

1. there is a well-defined date of cohabitation with current partner;
2. the couple are interested in getting pregnant as soon as possible after they cohabit; and
3. exposure to risk of pregnancy started on the date of cohabitation with current partner.

**FIGURE 2.**
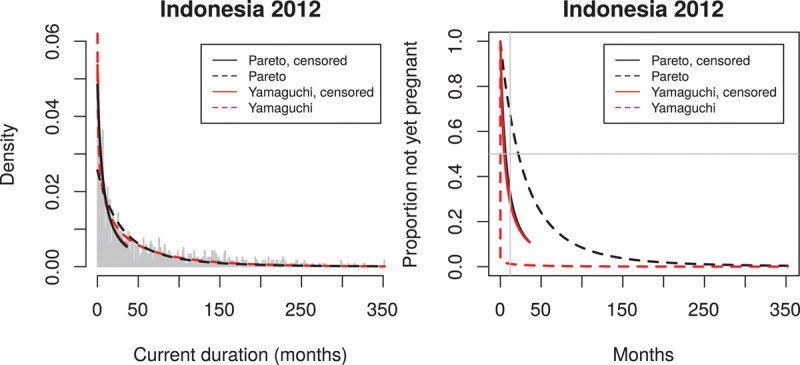
A: Fit of the CD distribution. Broken lines: estimated densities gP(y) and gY(y) based on all 1183 observations; the estimate of gY(0) = 1.75 while estimated gP(0) = 0.27. Fully drawn lines: estimated densities gP(y) and gY(y) based on all 1183 observations, but censoring the 538 observations larger than 36 months at 36 months. B: Estimated survival functions for times to pregnancy (TTP) based on the fits in Fig. 2A.

Figure 2A is a histogram of all 1185 observed CDs *y* from Indonesia 2012 with fitted Pareto and Yamaguchi probability densities *g*_*P*_(*y*) and *g*_*Y*_(*y*). It is seen that the pragmatic definition of waiting time to become pregnant results in observed values up to 360 months. The broken lines show the fits of *g*_*P*_(*y*) *and g*_*Y*_(*y*)—these fits are almost identical except close to 0 where we get estimated *g*_*Y*_(0) = 1.75 while estimated *g*_*P*_(0) = 0.27.

Figure 2B shows the estimated survival functions (broken lines)


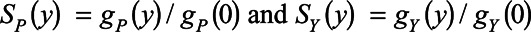


which show a dramatic difference mostly due to the different fits of *g*_*P*_(0) and *g*_*Y*_(0).

To focus on the shorter CDs of most interest in this application, we also followed established practice in the literature (as mentioned above) by studying the distribution *censored* at 36 months, that is, keeping the observations ≤36 months but replacing all observations >36 months by the knowledge that they are >36 months. The resulting fits (fully drawn lines in Figure 2A and B) are now very similar for Pareto and Yamaguchi, and the resulting estimates of the survival function of TTP provide the realistic estimates of median TTP = 4.9 months and estimated probability of not having become pregnant (as infertility is defined) at 12 months = 0.29.

**FIGURE 3.**
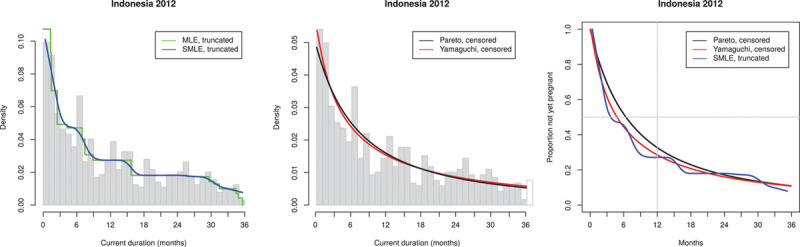
Observed current durations and estimated TTP distribution for Indonesia 2012 (1183 observations, of which 645 ≤ 36 months). Acceptable histogram. TTP, times to pregnancy.

To assess and supplement the fits of the two parametric distributions, we also used nonparametric fits to the CD distribution as described above. These are available for the distribution of CDs *truncated* at 36 months (i.e., the conditional distribution of CD, given it is ≤36 months). These fits are shown in Figure 3A. It is known that the MLE but not the SMLE of *g*(*y*) is inconsistent at 0, and because the survival function of TTP is obtained by division by the estimated *g*(*0*), we therefore only use the SMLE in the further calculations.

In the following, we restrict attention to the common standard of analyzing CDs censored (for nonparametric fits: truncated) at 36 months.

As mentioned earlier, the analyses are based on CDs of the pregnancy attempt for each nulliparous woman, obtained from all women who met the inclusion criteria as how long before the interview she had initiated her attempt to get pregnant, in practice at the most recent establishment of a relation with a male partner.

We show below the results for all 15 DHS data sets in the same layout based on triples of diagrams as illustrated in Figure 1 for the data from the ObsEFF study: to the left, the histogram (truncated at 36 months) of the observed CD, with nonparametric fits to the observed CD as specified above (Groeneboom MLE, truncated and Groeneboom SMLE, truncated), in the middle, the parametric fits to observed CD (Pareto, censored at 36 months and Yamaguchi, censored at 36 months); to the right, the consequent estimates of the survival function of TTP for all of these except the MLE which is known to be biased.

### Observed Histograms Have too Few Observations Near the Origin

**FIGURE 4.**
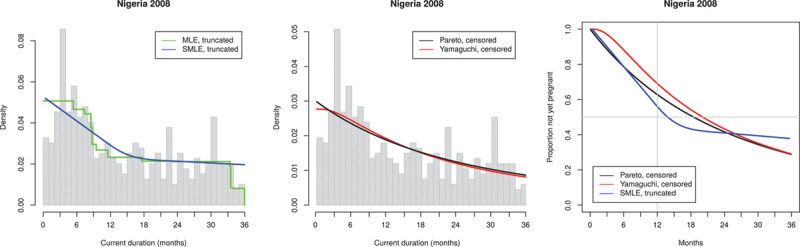
Observed current durations and estimated TTP distribution for Nigeria 2008 (683 observations, of which 397 ≤ 36 months). Irregular histogram. TTP, times to pregnancy.

**FIGURE 5.**
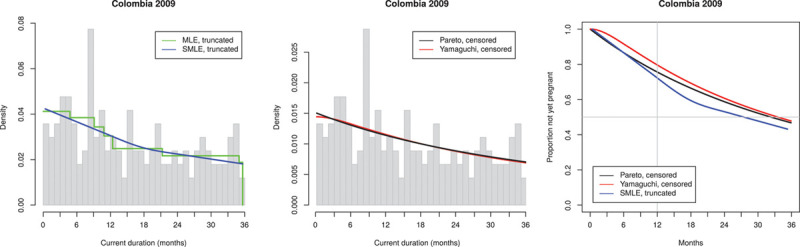
Observed current durations and estimated TTP distribution for Colombia 2009 (456 observations, of which 168 ≤ 36 months). Flat histogram. TTP, times to pregnancy.

Returning to the DHS-based results, we focus on the variation in the structure of the observed histograms of CDs. We recall that in theory the observed histogram should be decreasing (being an estimate of the density function of CDs which in theory is proportional to the decreasing survival function of TTP). We divided our results according to characteristic patterns illustrated by Indonesia 2012 (Figure 3) where the histogram is close to decreasing, we categorize this as acceptable, Nigeria 2008 (Figure 4) with clear deviation from the expected decreasing shape, we categorize this as irregular and Colombia 2009 (Figure 5), which is actually closer to being constant than decreasing; we categorize this as flat.

All 15 data sets are sorted into the three categories Online Supplementary Material; http://links.lww.com/EDE/B742. A main feature is that the more flexible Yamaguchi distribution usually corresponds better than the Pareto distribution to the nonparametric fits. Further descriptions of the three categories are given in the following.

From the estimated TTP distribution, one may directly derive estimates of *median* TTP (months) and estimated per cent not yet pregnant after 12 months of trying, the so-called infertility, see Table 2.

**TABLE 2.** Estimated Median TTP and Probability of Not Having Conceived Within 1 Year (So-called infertility) for All 15 Test Data Sets, Based on Fitting Yamaguchi Distributions to the Current Durations Censored at 36 Months

	Number		Median TTP (Months)			Estimated Infertility (%)			% Pregnant by Duration of Cohabitation					
Subjective allocation of histograms			Acceptable	Irregular	Flat	Acceptable	Irregular	Flat	Acceptable		Irregular		Flat	
Months cohabitating									0–2	3–5	0–2	3–5	0–2	3–5
	Total	Uncens at 36 months												
Benin 2006	250	139	8.1			0.38			23.1	51.2				
Colombia 2009^a^	452	168			33.6			0.80					24.7	39.4
Colombia 2016	316	114			≥36			0.85					30.4	36.3
Dominican Republic 2002	243	134			17.4			0.56					23.7	34.1
Dominican Republic 2007	245	116			27.1			0.75					29.4	27.0
Indonesia 2002	1005	517	5.0			0.28			15.1	45.3				
Indonesia 2007	1076	562	4.6			0.26			17.3	46.8				
Indonesia 2012^a^	1183	645	4.8			0.28			19.8	46.2				
Nigeria 2008^a^	670	397		20.1			0.69				37.3	40.8		
Nigeria 2013	756	432	5.1			0.28			21.3	45.5				
Phillipines 2003	315	139	10.5			0.44			42.2	52.7				
Phillipines 2008	271	133	3.6			0.24			25.5	48.4				
Senegal 2005	234	112		8.5			0.41				0.0	33.3		
Senegal 2010	248	120		12.5			0.51				4.8	34.5		
Tanzania 2015	211	151		13.0			0.55				25.0	41.0		

Also displayed: % pregnant by duration of cohabitation (data from DHS).

No correction for delayed pregnancy recognition has been attempted.

^a^Examples displayed as Figures 3–5.

DHS indicates Demographic and Health Surveys; TTP, times to pregnancy.

The seven histograms characterized as acceptable—all from Africa and Asia (e.g., Indonesia 2012 [Figure 3]). All display a reasonably decreasing shape as predicted by the theory of the CD approach. The estimation of median TTP ranges from 3.7 to 10.5 months (without correction for delayed recognition of pregnancy) and estimated infertility prevalence ranges from 24% to 44%.

The four irregular histograms, all from Africa (e.g., Nigeria 2008 [Figure 4]) display rather dramatic variation generating unrealistic estimates: the median TTP is estimated as 12.5–20.9 months (without correction for delayed recognition of pregnancy) and the estimated infertility prevalence ranges from 51% to 69%.

The four flat histograms, all from Latin America (e.g., Colombia 2009 [Figure 5]) are rather similar among themselves: wildly varying with only a minor downward trend, far from the theoretical decreasing shape. The median TTP is estimated as 19.6–33.5 months (without correction for delayed recognition of pregnancy) for the three data sets where the median is estimable (for Colombia 2016, the probability of not having conceived at the maximum included time of 36 months is larger than 50% so that we can only state that the median is estimated as being >36 months); the estimated infertility prevalence ranges from 58% to 94%.

#### Unexpected Shape of Histograms

The nonmonotonicity of the histograms is a major problem. Except for most of the acceptable histograms, there are too few women on interview who recently entered into cohabitation with a man. As explained earlier, this nonmonotonicity indicates serious departure from the assumption of constant rate over calendar time of initiating attempts to become pregnant. We return below to discussion of possible explanations of this bias which may be generated by the sample selection procedure of DHS and/or selective attrition among women who were recently married (or had otherwise entered cohabitation with a man). Table 2 includes information on the frequency of pregnant women among DHS participants who recently established cohabitation.

It should be emphasized that the empirical tendency of too few CDs near zero cannot be explained by pregnancy recognition delay.

It is obvious that the flat histograms and several of the irregular histograms lead to unrealistically high values of median TTP as well as infertility. It should here be remembered that no correction has been attempted for the likely pregnancy recognition bias. However, from subject matter considerations, this should not realistically exceed 4–5 months, and Table 2 shows much larger deviations from realistically expected values.

## DISCUSSION

In this feasibility study of the possible use of the CD approach to monitoring infertility based on the DHS, we have restricted attention to nulliparous women. We have seen that the key pattern of observed CDs since the chosen proxy^17^ for the initiation of pregnancy attempt deviates considerably from the assumptions necessary for the estimation of the distribution of TTP. We also need to recall that we did not have access to information on possible infertility treatment.

It seems obvious that it is a too crude approximation to consider the most recent entrance into cohabitation as proxy of the initiation time of attempts to get pregnant.

An important difficulty here is that use of contraception is only recorded at time of interview, so that possible use of contraception from entrance into cohabitation until some time before the interview is mistakenly included in the registered CD, precisely generating the observed lack of short CDs. (A similar false effect would come from possible miscarriages or stillbirths which would prolong the time where the woman is still technically nulliparous.)

The lack of short CDs is particularly apparent for Colombia and the Dominican Republic (both countries with flat histograms), well in agreement with the much higher prevalence of use of contraception in Latin America than in Western Africa. (In agreement with this, comparison of the columns S4 and S5 of Table 1 for Colombia and the Dominican Republic indicates particularly high prevalence of contraception among all interviewed women.)

Another contributing reason could be that the method ignores that pregnancies among separately living couples may lead to establishment of cohabitation rather than the other way round. Pregnancies happening before cohabitation will decrease the pool of newly cohabiting nonpregnant nulliparous women, violating the crucial assumption of independence between recruitment of nullipari and getting pregnant. We have scant information about this event from DHS, although Table 2 shows a (weak) tendency that the percentage of pregnant nullipari women right after the start of cohabitation is larger in the countries with flat histograms than in those with acceptable histograms.

A further important issue is whether there are inhomogeneities in the recruitment pattern (or the compliance) of the DHS that might help explain the observed irregularities.

We have to conclude that further work on using CD methodology in connection with DHS should start by investigating whether it would be feasible to include a direct question about initiation of pregnancy attempt, as in existing French and the United States^13,18^ experience. Such questions should of course be formulated carefully to be unobtrusive such as “When did you first start having intercourse without contraception?” or “How long have you been having intercourse without using a method to avoid pregnancy?”

## CONCLUSIONS

Our conclusion of this exercise is that the practice of estimating realistic CDs for nullipari under the assumption that initiation happens at the date of establishment of the relationship—does not work in a sufficiently broad context to make the present form of DHS data applicable for monitoring infertility via the CD approach. To allow for deriving valid estimates of infertility DHS should consider expanding their questionnaires to include more specific questions, including times of initiating attempts to become pregnant, in future DHS rounds, after piloting the proposed questions to assess their validity and reliability.

## ACKNOWLEDGMENTS

We thank Professor P. Groeneboom for helpful correspondence on nonparametric maximum likelihood estimates including sharing his software with us, and we thank Margit Schilling Riis for energetic computing assistance. We are very grateful for insightful and detailed comments from two referees.

## Supplementary Material


