# Supplementary material for: The Use of Time to Pregnancy for Estimating and Monitoring Human Fecundity From Demographic and Health Surveys
Source: Epidemiology. 2020 Nov 30;32(1):27–35. doi: 10.1097/EDE.0000000000001296 (PMC7707157; doi:10.1097/EDE.0000000000001296)
Supplement: Supplementary file 1 [file ede-32-027-s001.pdf]

The use of time-to-pregnancy for estimating and monitoring human fecundity from Demographic and Health Surveys (DHS)

Niels Keiding, Mohamed M. Ali, Frank Eriksson, Thabo Matsaseng, Igor Toskin, James Kiarie

Supplementary Digital Content

1. **Display of all 15 data sets with fitted TTP distribution**
2. **Results of simulation studies of the correction for possible pregnancy recognition bias proposed by Polis et al. (2017)**
3. **Availability of interview date**
4. **Comparison of our analysis with that of Polis et al. (2017)**

1. Display of all 15 data sets with fitted TTP distributions

The data are displayed in crude order of decreasing ‘acceptability’ of the shape of the histogram of current durations:: 7 ‘acceptable’, 4 ‘irregular’ and 4 ‘flat’.

7 Acceptable

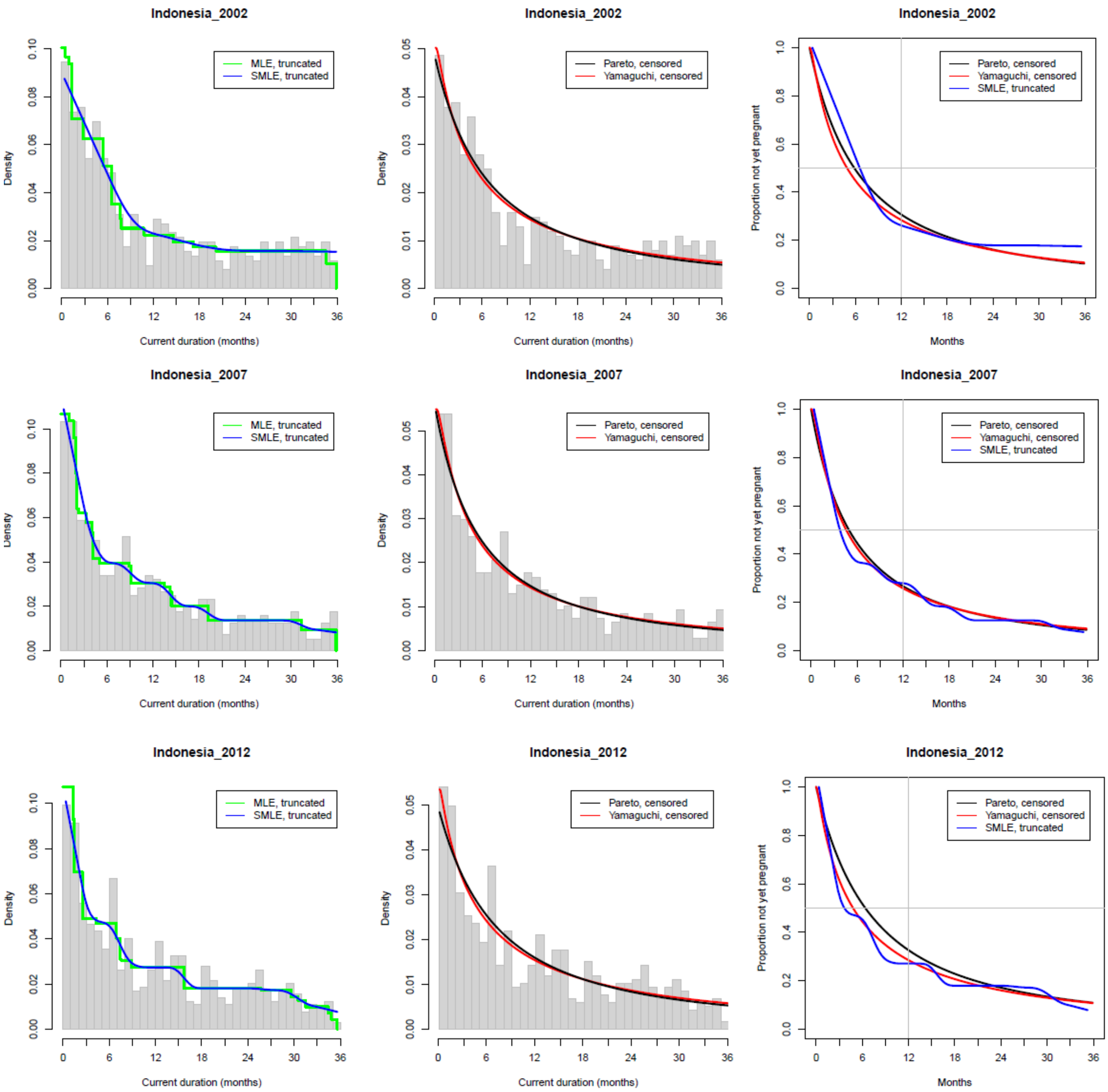

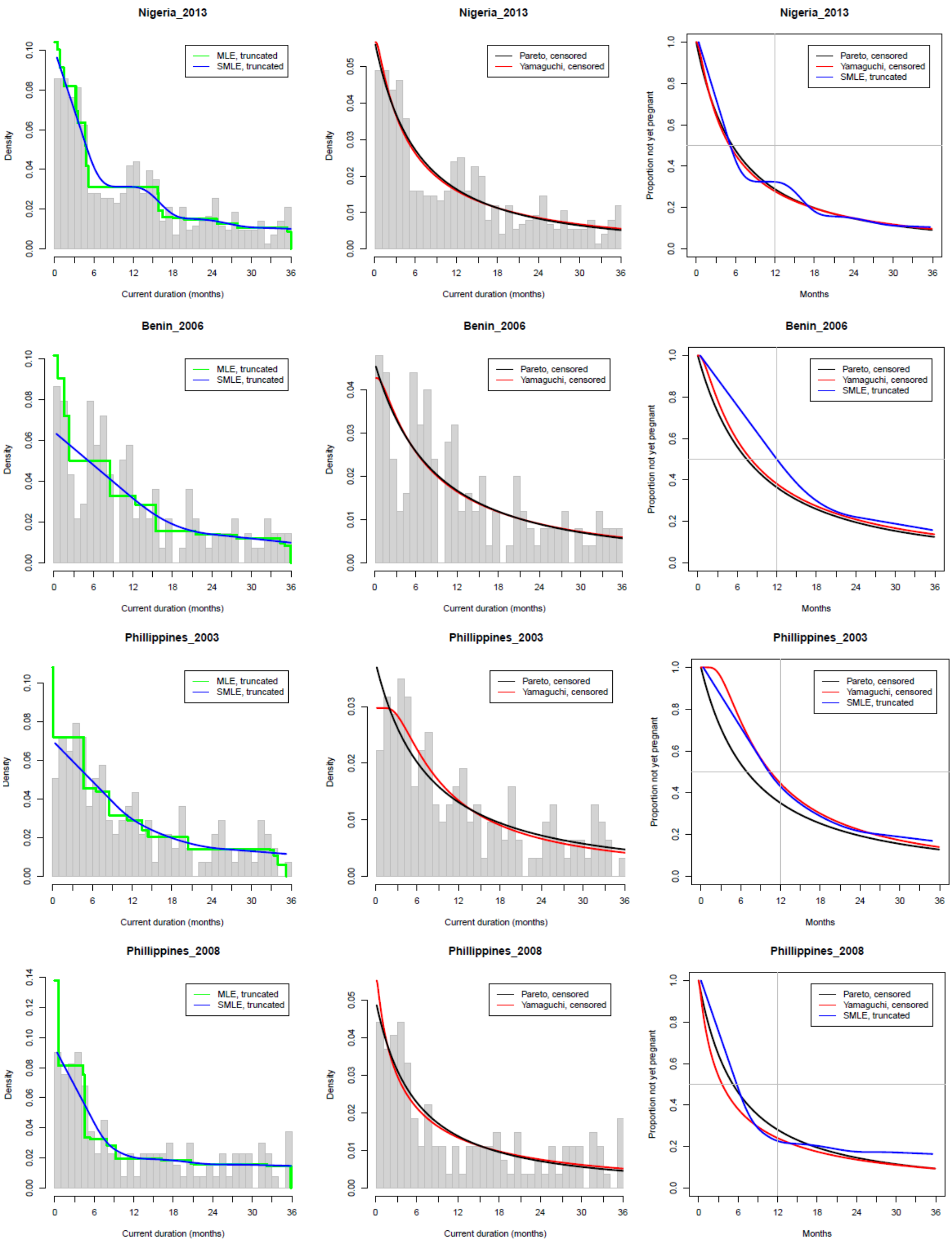

Nigeria_2013
MLE, truncated
SMLE, truncated
Pareto, censored
Yamaguchi, censored
Density
Current duration (months)
Proportion not yet pregnant
Months
Benin_2006
Phillippines_2003
Phillippines_2008

4 Irregular

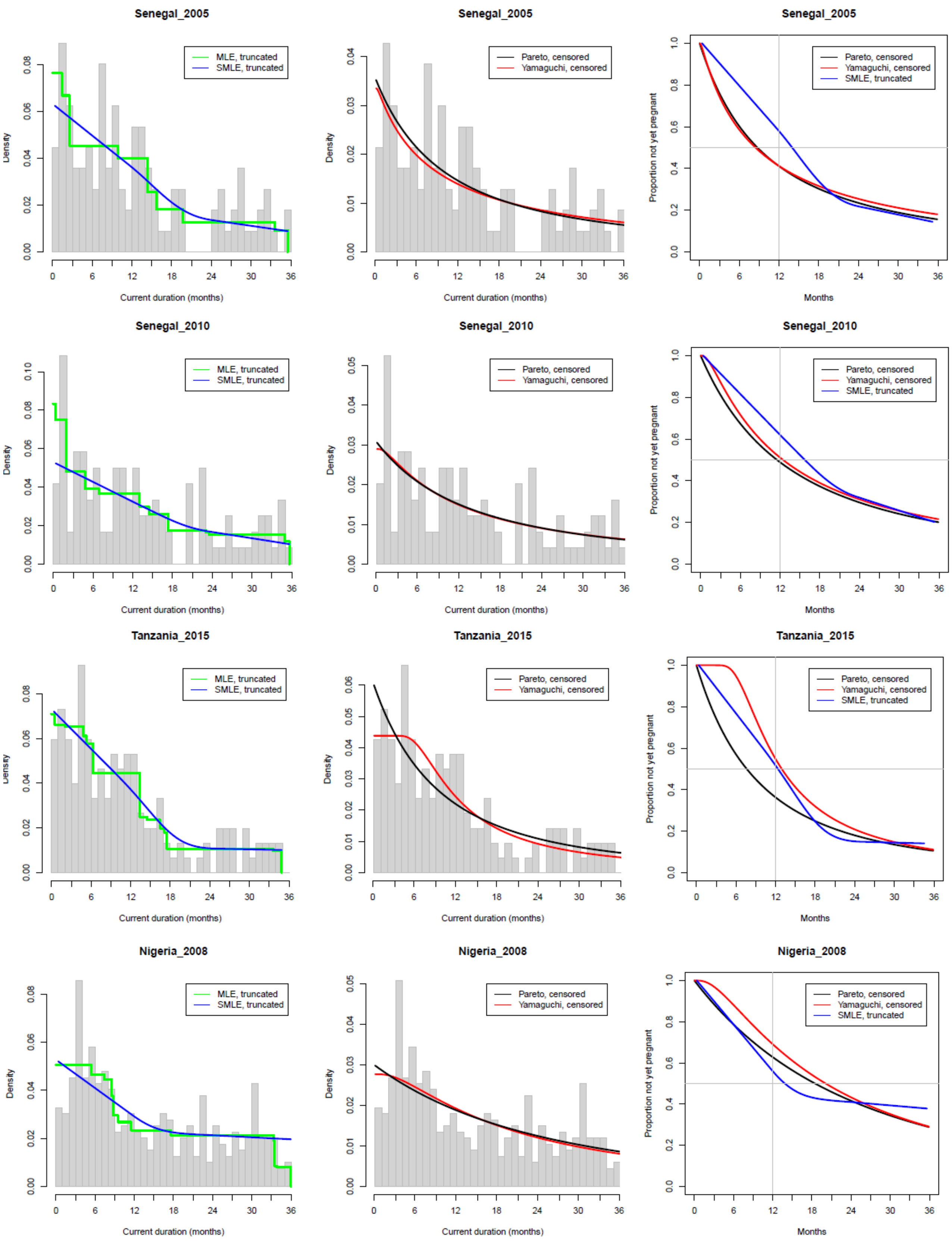
Senegal_2005
Senegal_2010
Tanzania_2015
Nigeria_2008
MLE, truncated
SMLE, truncated
Pareto, censored
Yamaguchi, censored
Density
Current duration (months)
Proportion not yet pregnant
Months

4 Flat

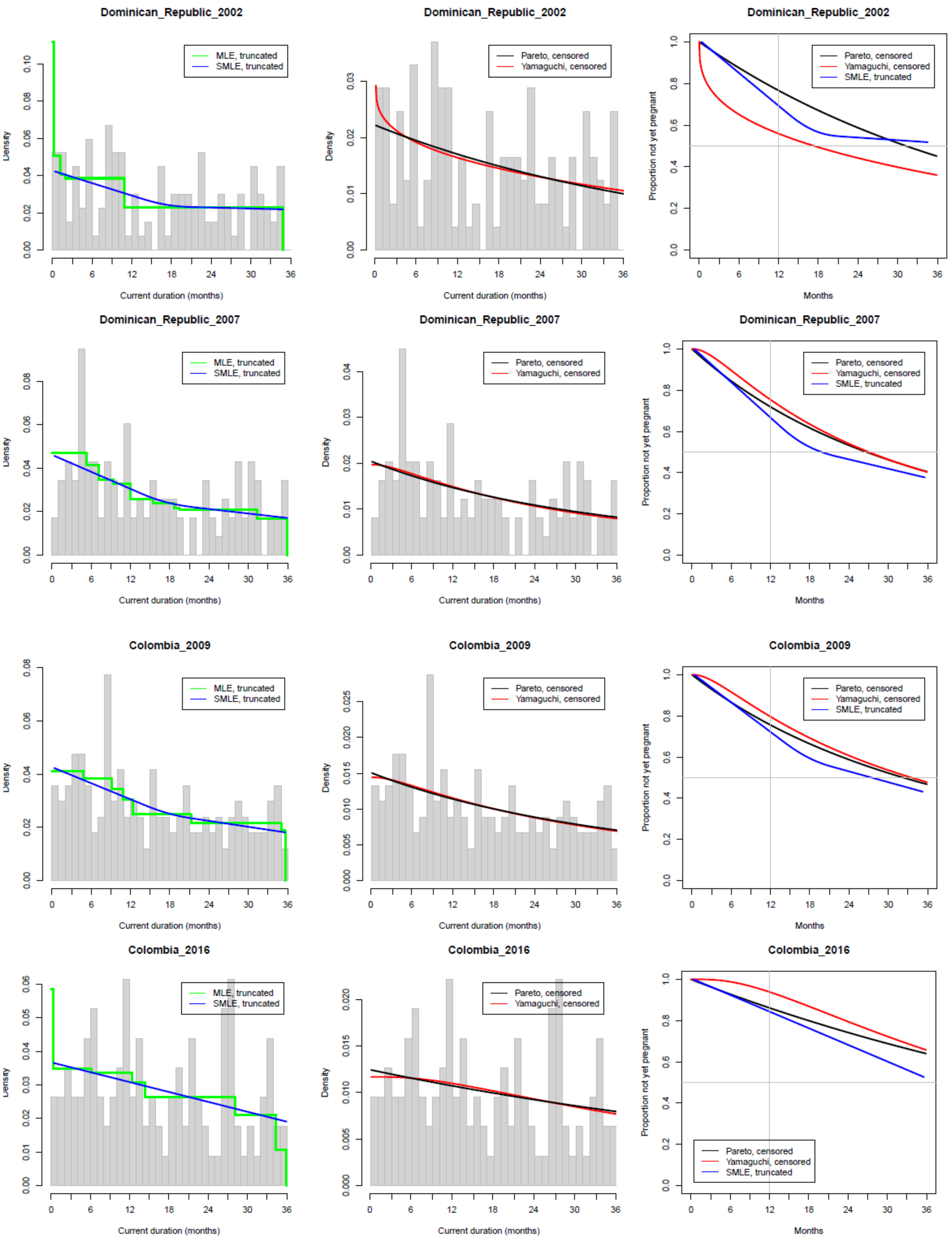
Dominican_Republic_2002
MLE, truncated
SMLE, truncated
Pareto, censored
Yamaguchi, censored
SMLE, truncated
Density
Proportion not yet pregnant
Current duration (months)
Months
Dominican_Republic_2007
Colombia_2009
Colombia_2016

2. **Results of simulation studies of the correction for possible pregnancy recognition bias proposed by Polis et al. (2017)**

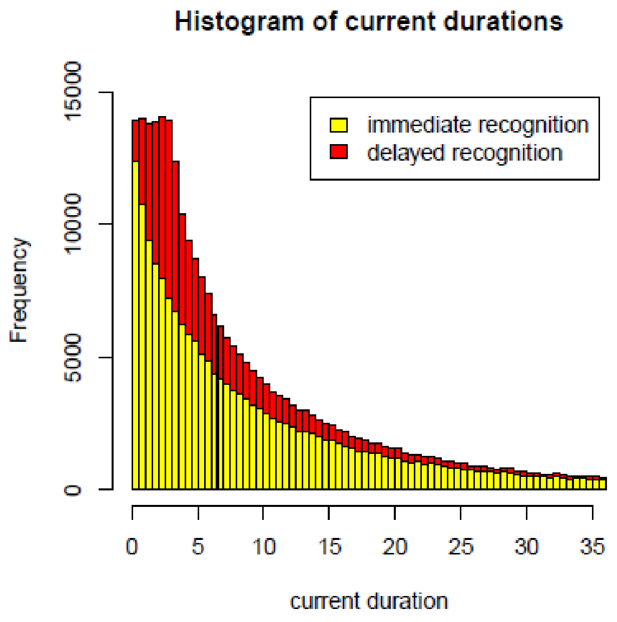

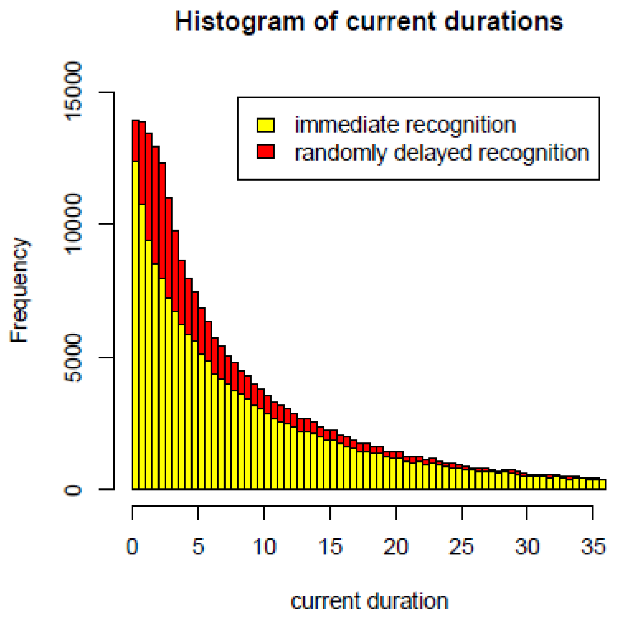

eFig. 1a (Left). Current durations CD_{obs}, observed if all pregnancies are first recognized 3 months after they happened (red). $CD_1 = CD_{obs} - 3$ months is in this case identical to the histogram of current durations obtained if all pregnancies were immediately recognized (yellow).

eFig. 1b (Right) Current durations CD_{obs}, observed if pregnancies are recognized with linearly increasing probability across the interval [0 months, 3 months] after they happened (red). In this case the histogram of CD_{obs} bears no simple relation to the histogram of current durations obtained if all pregnancies were immediately recognized (yellow).

3. Availability of interview date

A good part of our work with these data took place under the assumption that only the month, not the date of the interview was available in the public domain-version of the DHS results. It has later turned out that the precise date is actually available but since there are rich possibilities for bias if these issues are ignored we give here a brief summary of our analyses.

We note below that Polis et al. (2017) did not use the precise date of the interview, but only the month.

Construction of initiation date on the assumption that the precise date of the interview is unavailable

A key component of the current duration approach is the existence of a precisely registered date of initiation of the pregnancy attempt. In the DHS this is not part of the routine, so a construction is applied; here we *focus on childless women* and assume that initiation takes place at the date of start of the current relationship: either date of marriage or date of establishing the relationship. The typical question is not about the date but about the month where the relationship started:

Did your relationship start *this month / last month / two months ago /… ?*

Rounding initiation and interview dates

We assume that woman i has precise continuously varying initiation date s_i and interview date t_i so that the current duration $y_i = t_i - s_i$.

We only know the interview *month* and assume that t_i is uniformly distributed in that month with mean at mid-month.

The women is asked

Did your relationship start
this month / last month / two months ago /… ?

Without further assumptions we will have, given t_i

this month: s_i uniform across ($[t_i]$, t_i) and therefore

$y_i = t_i - s_i$ uniform across (0, $t_i - [t_i]$) with mean ½($t_i - [t_i]$) and marginal mean ¼.

last month: s_i uniform across ($[t_i-1]$, $[t_i]$) and therefore
$y_i = t_i - s_i$ uniform across ($t_i - [t_i]$, $t_i - [t_i - 1]$) with mean $t_i - [t_i] + ½$ and marginal mean 1.

two months ago: s_i uniform across ($[t_i - 2]$, $[t_i - 1]$) and therefore

$y_i = t_i - s_i$ uniform across ($t_i - [t_i - 1]$, $t_i - [t_i - 2]$) with mean $t_i - [t_i] + 1 ½$ and marginal mean 2.

From these calculations it is clear that on average (or assuming interview happens at mid-month), blue intervals to be explained below.

this month represents current durations in (0,0.5] (0.1]

last month represents current durations in (0.5,1.5] (1,2]

two months ago represents current durations in (1.5,2.5] (2,3]

etc.

However, in our initial analyses we understood the data as representing the blue intervals. Fig. 1 based on simulated Pareto distributed TTP shows that this interpretation does generate 'too few' small current durations. A fit of the Yamaguchi density function to the current durations is included.

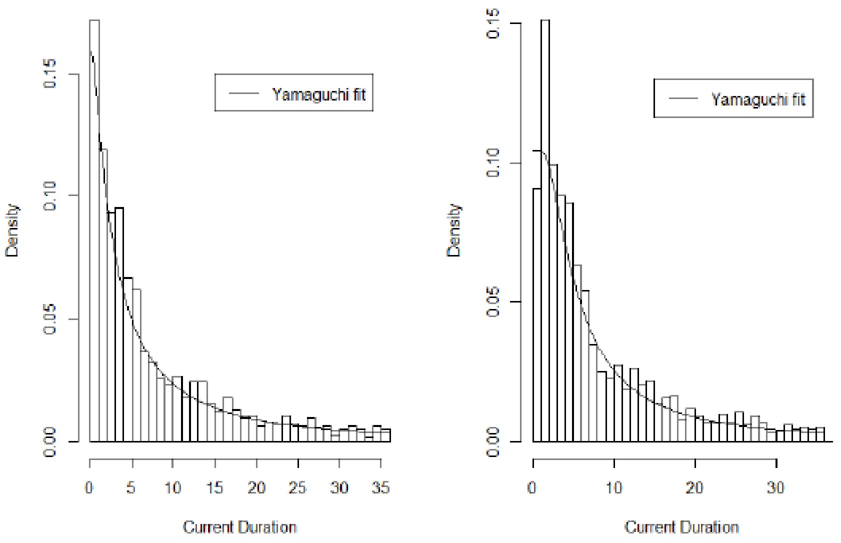

Fig. 1. 1736 current durations generated by Pareto distributed TTP with mean 6.2 and median 2.74.
Left: standard histogram; Right: histogram based on the misinterpretation 'blue intervals'

Fig. 2 compares various estimates of the survival curve of the TTP, including the true Pareto distribution underlying the simulation, and Table 1 shows that a dramatic overestimation of the median TTP results from the misinterpreted roundings.

'Correct' coding when interview date is available

When the date of the interview is available (but only month is available for start of cohabitation) we calculate current duration as the time between the date of the interview and

1. Day 15 of the month of start of cohabitation, unless this month is the same as the interview

2. The midpoint between the start of the current month and the interview date, if the interview took place in the month of start of cohabitation.

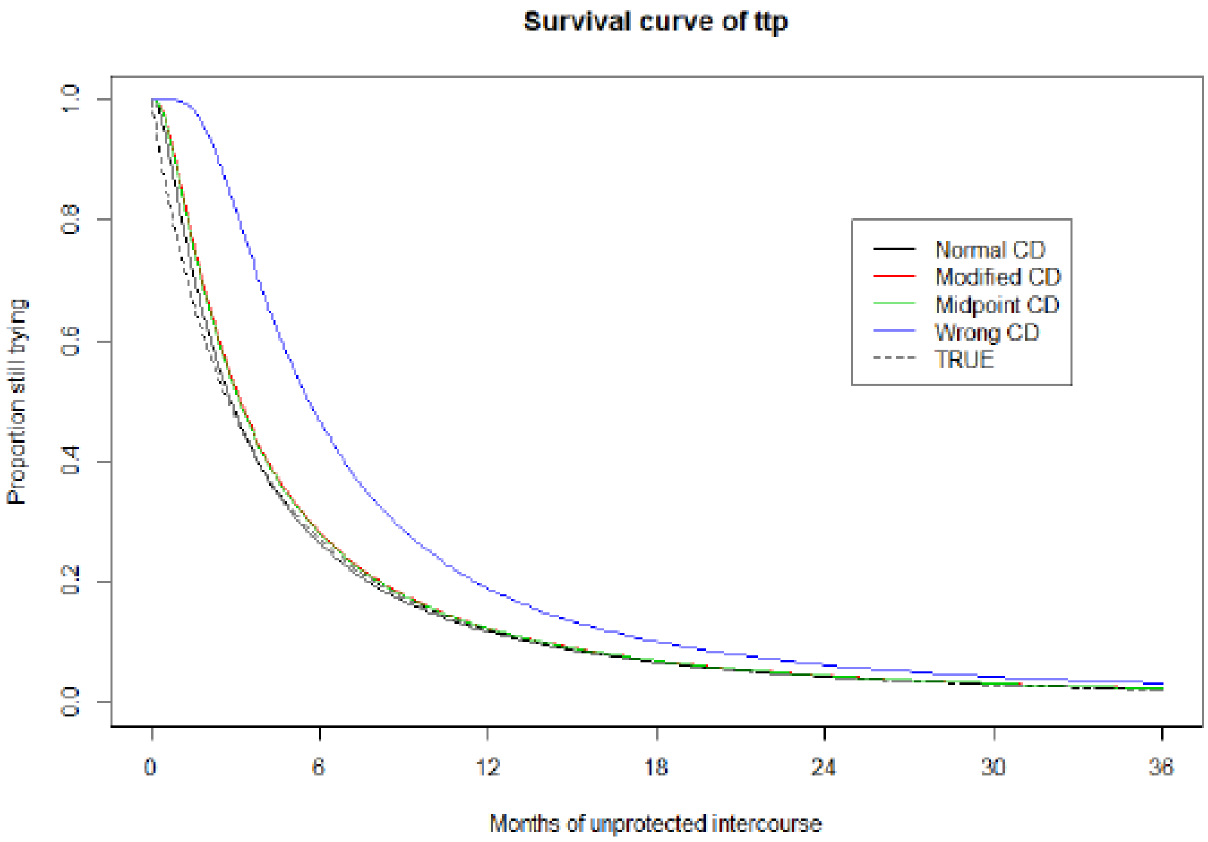

Fig. 2. Pareto distributed TTP. Estimated survival curves for TTP based on Yamaguchi fit to various interpretations of the 'current duration' histograms.

····· true survival function ––––– direct, continuous time ––––– correct, using midpoints

––– misunderstanding: mass from (0,0.5] filling (0,1] , (0.5,1.5] filling (1,2] etc.

––––– modified (correctly allocated bars, using midpoints)

Current duration type	continuous	modified	midpoint	wrong	TRUE
Median	2.85	3.15	3.15	5.65	2.75

Table 1. Pareto distributed TTP. Estimated median TTP. The apparently innocent 'misunderstanding' generates an overestimation of more than 100% of the median TTP.

‘Repairing’ the DHS data?

The above simulation study demonstrates that the above misunderstood construction of initiation date may have a rather dramatic effect on the estimated TTP distribution. Since a major issue is the mistaken interpretation of the frequency across [0,1] (the blue interval above) as covering a full month rather than a half month we briefly explored whether a simple solution replacing the reported intervals (0,1], (1,2], … by (0,½), (½,1½], (1½,2½], … would repair the problem.

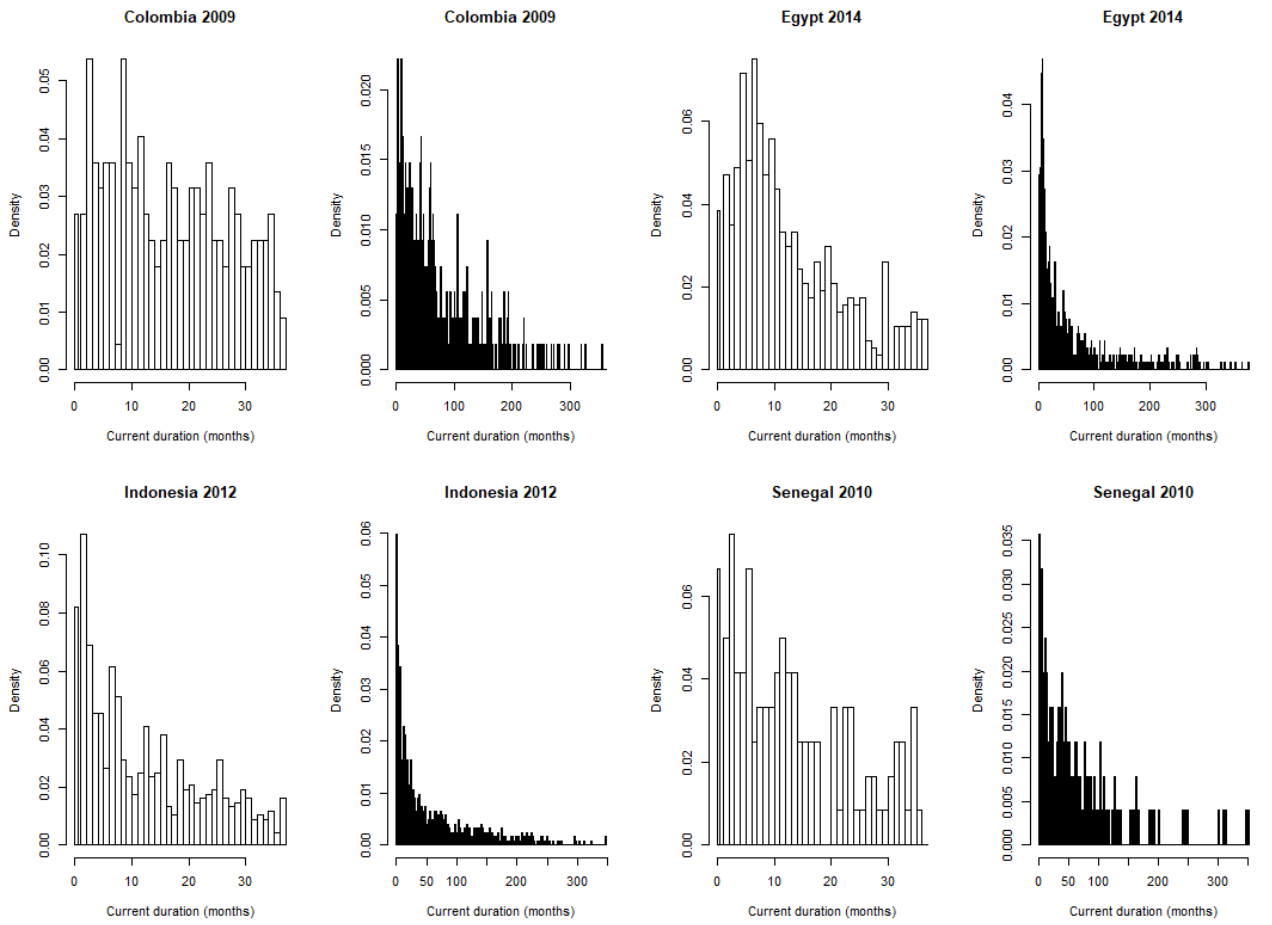

Fig. 3. Modified histograms, replacing (0,1] by (0,½] (so that the frequency doubles), and all other (i,i+1) by (i − ½, i+½) .

Fig. 3 shows the change for four of the rather problematic countries, with the first frequency no longer so dramatically ‘too short’. The effect on estimating the TTP distribution is shown in Fig. 4 and Table 2. Although an important modification is obtained for Indonesia 2012, the other modifications do not bring the median TTP within realistic range (all estimates being larger than one year).

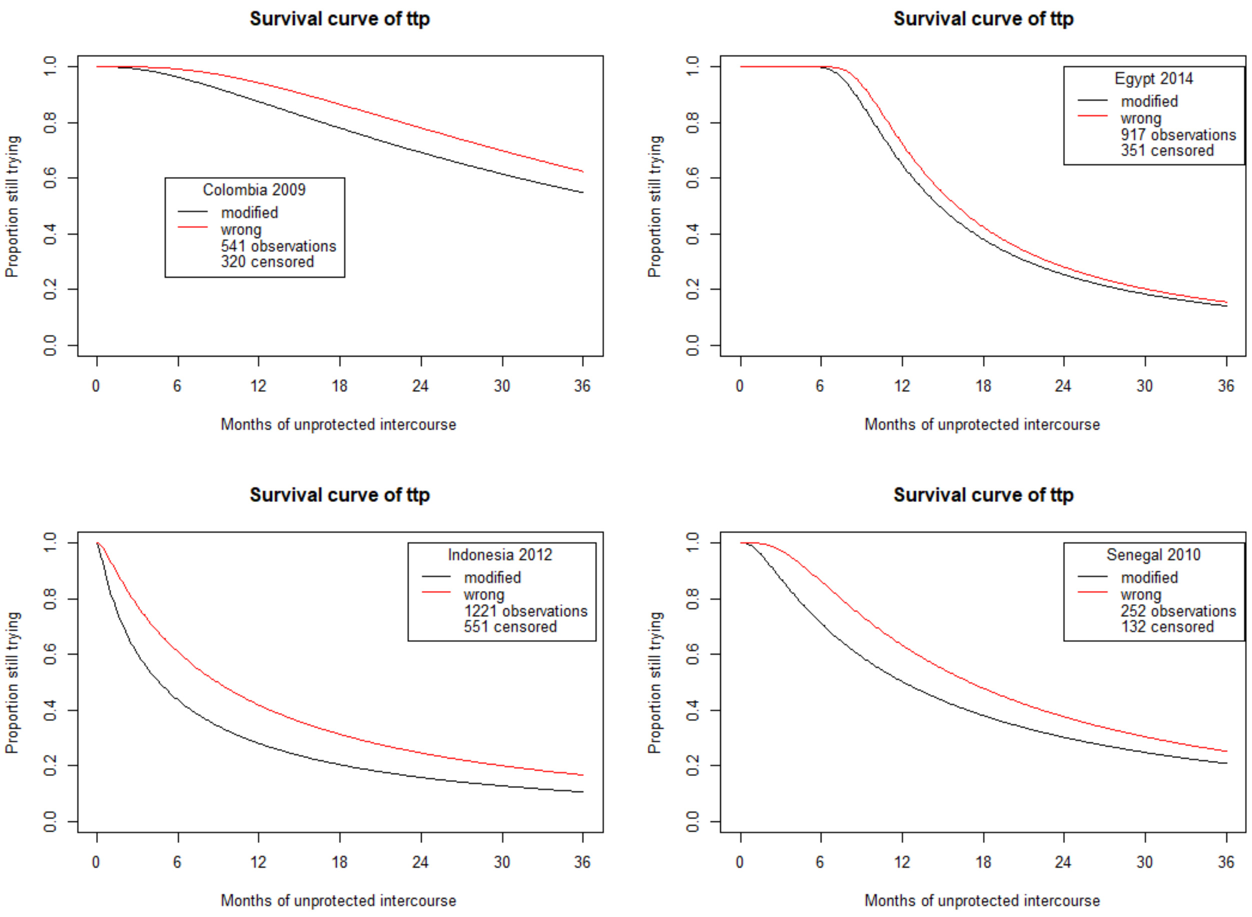

Fig. 4. Estimated survival curves of TTP generated from Yamaguchi fits to the modified 'current durations displayed in Fig. 3. The red curves represent fits based on the originally received data and the black curves the result of the modified interpretation of the short 'current durations' explained above.

	Modified	Wrong
Egypt	14.8	16.1
Indonesia	4.7	9.0
Senegal	12.1	17.1

Table 2. Estimated median TTP(months) for the data displayed in Fig.3.7.

Note that Colombia has estimated medians > 36 months before as well as after modification.

Conclusion on the bias of initiation date

The analysis shows that the above described incorrect construction of initiation date does generate a significant bias. In principle it should be possible to counteract most of the effect of this bias by reinterpreting ('repairing') the data, but as judged from the estimate of median TTP this device still does not generate credible results for several countries.

Polis et al. (2017)

We note that Polis et al. (2017) did not have access to the precise date of interview and did not discuss this issue explicitly in their paper. In private communication, April 2019, co-author Marie Thoma explained that

The R code shows that values of 0 were adjusted to 0.25 as done in other applications of this approach

and this is confirmed in the published software documentation of Polis et al. Thus it seems that Polis et al. actually used the same interpretation as the one we termed 'modified' above.

4. **Comparison of our analysis with that of Polis et al. (2017)**

Our analysis generally followed the same choices as that of Polis et al. (2017). Thus the selection of participants from each single DHS survey followed the same principles, as we carefully verified through detailed correspondence with Marie Thoma (University of Maryland, USA, co-author of Polis et al. (2017)) for the data from Nigeria 2013 analysed by Polis et al.

There were two exceptions:

a. As explained in the main text our analysis of Polis et al.s idea of omitting the first three months concluded in not supporting this route to handling pregnancy recognition delay, so we did not perform that truncation of the data.
b. Polis et al. worked from a version of the data which contained only the month of interview, not the precise date. For a long time we were under the same impression and therefore worried about the best approximation. After our further scrutiny revealed that the precise date is actually available in the public domain version of DHS, we used that throughout. As mentioned above, Polis et al. 'adjusted values of 0 to 0.25' which according to our detailed analysis is the best thing to do if the precise date is not available.
